# Assessment for late developmental hip dysplasia in a cohort of infants with risk factors and normal hip ultrasound

**DOI:** 10.3389/fped.2023.1140349

**Published:** 2023-03-21

**Authors:** Rosa Morello, Beatrice Bocchi, Francesco Mariani, Alice Bononi, Cristina Giuli, Nadia Bonfiglio, Piero Valentini, Ilaria Lazzareschi, Claudia Rendeli, Osvaldo Palmacci, Danilo Buonsenso

**Affiliations:** ^1^ Department of Woman and Child Health and Public Health, Fondazione Policlinico Universitario A. Gemelli IRCCS, Rome, Italy; ^2^ Department of Orthopedics, Fondazione Policlinico Universitario A. Gemelli IRCCS, Rome, Italy; ^3^ Department of Orthopedics, Università Cattolica del Sacro Cuore, Roma, Italy; ^4^ Department of Woman and Child Health and Public Health, Medical School, Università Cattolica del Sacro Cuore, Roma, Italy; ^5^ Centro di Salute Globale, Università Cattolica del Sacro Cuore, Roma, Italy

**Keywords:** DDH, hip ultrasound, Graf technique, late DDH, risk factors, breech position

## Abstract

**Background:**

Developmental dysplasia of the hip (DDH) is a known orthopedic pathology of newborns that, if not diagnosed and treated, can lead to debilitating long-term consequences. Ultrasound has proven to be an effective method for the early diagnosis of this condition. Recently, reports of late DDH in populations at risk (breech presentation) and after negative ultrasound examination have emerged in the literature.

**Aim:**

The objective of the study was to assess the possible appearance of late DDH in Italian children with risk factors but negative ultrasound screening.

**Materials and methods:**

We selected patients with risk factors for DDH and a negative hip ultrasound from the medical records of children referred to the Hip Ultrasound Clinic (Rome, Italy) from January 2018 to November 2021. To identify possible cases of late DDH, from February 2022 to July 2022, all patients who met the inclusion criteria were submitted to orthopedic follow-up clinical evaluation. In the case of a pathological objective examination, radiography was performed.

**Results:**

Fifty-five patients (52.7% female, 52.7% with breech presentation, and 41.8% with a positive family history) met the inclusion criteria. The median age of gait onset was 13 months. The median age of orthopedic follow-up examination was 45 months. Only three patients (5.5%) had a pathological examination, but no x-ray were pathological.

**Conclusion:**

Our study has not documented cases of late DDH. Considering the small study population and the only clinical follow-up, further studies are needed to clarify the possible late development of this condition.

## 1. Introduction

Developmental dysplasia of the hip (DDH) is one of the most common musculoskeletal disorders of newborns, with incidence estimates of 4.4–23.0/1,000 live births (1). DDH is a complex and dynamic process that encompasses a broad spectrum of developmental abnormalities of the hip, varying from mild dysplasia to complete hip dislocation (2). Although the exact pathophysiological mechanisms are still unknown, there are several known risk factors for DDH. The most important single risk factor is breech position, followed by the female sex, incorrect lower-extremity swaddling, and a positive family history. Other suggested, but not documented, findings are being the first-born, torticollis, foot abnormalities, oligohydramnios, multiple gestations, and macrosomia (3, 4).

Because hip development disorders have the potential to cause long-term disabling complications such as gait abnormalities, muscle hypotrophy, degenerative hip, knee injuries, and chronic pain, the main goal is early diagnosis and treatment (5). Moreover, early detection and referral of newborns with DDH could allow for conservative intervention, preventing the need for surgery (6). About conservative management of DDH, depending on the patient's age and the severity of the disease, the dynamic or static splint is actually considered the gold standard treatment. In particular, in cases of instability and dislocation, a dynamic splint is the most commonly used treatment, especially if applied within 4–5 months of life. The Pavlik harness appears to be the most used brace (7). According to medical literature, despite the current debate between selective and universal screening (8–10), hip ultrasound is considered the most reliable diagnostic examination for DDH (11, 12). Until now, available literature (13–15) did not recommend (even in children with risk factors, including breech presentation) further follow-up after a negative hip ultrasound examination.

Recently, literature has highlighted the possibility of a “late DDH” diagnosis after a negative initial ultrasound screening in children with breech presentation (16–22). If this condition were to be confirmed, there would be a group of children for whom current screening programs might not be sufficient. Therefore, for some patients, it would be necessary to carry out subsequent follow-ups after a negative ultrasound screening. However currently, there are still doubts about this condition and its incidence, as well as the timing and modalities of follow-up (clinical vs. radiological) (23).

Given this evidence, we conducted a study to evaluate, in our population, the possible impact of late hip dysplasia in children with risk factors for DDH and negative ultrasound screening.

## 2. Materials and methods

### 2.1. Study population and setting

The study was conducted at the Hip Ultrasound Clinic and Pediatric Orthopedic Clinic in a tertiary hospital (Policlinico Universitario A. Gemelli, Rome, Italy).

The authors analyzed retrospectively the medical records of children referred to the Hip Ultrasound Clinic from January 2018 to November 2021. The following information has been acquired for each patient: medical history (date of birth, sex, gestational age, delivery, risk factors for DDH), date of hip ultrasound screening, medical report (normal vs. pathological), and Graf classification (for both right and left hip).

For our aim, was carried out a follow-up evaluation of patients who met the following inclusion criteria:

– Children who had performed hip ultrasound at our Hip Ultrasound Clinic during the study period
– Patients with at least one risk factor for DDH
– Negative ultrasound examination screening
– Children who had already learned to walk at the time of the research
– The parent's/caregiver's/guardian's consent to participate

The following exclusion criteria were applied for the study:

– Children who had performed hip ultrasound at our Hip Ultrasound Clinic outside the study period
– Patients without risk factors for DDH
– Patients with a pathological ultrasound examination screening
– Children who had not yet learned to walk at the time of the research
– Children without parents/legal guardian consent

About risk factors, the authors considered both the most established findings such as breech position, female sex, and positive family history, as well as other related features such as the first-born, torticollis, foot abnormalities, oligohydramnios, multiple gestations, and macrosomia.

### 2.2. Hip ultrasound examination

Hip ultrasounds were performed by one certified (and with several years of experience) pediatrician (DB), who participated to a Hip Ultrasound Training by Graf. A 12 MHz linear probe (EsaoteMyLab 40) was used. According to Graf methods, the correct standard section was obtained through coronal scans of each hip (a minimum of two sonograms) with a child in the lateral decubitus position (24). A morphological evaluation (presentation of lower limp, chondro-osseous border, femoral head, acetabular labrum, bony roof, bony rim and exclusion of tilting errors) and quantitative evaluation (by calculation of alpha and beta angles) were performed for each ultrasound examination (25).

### 2.3. Follow-up evaluation

To identify possible cases of late DDH, from February 2022 to July 2022, all patients who met the inclusion criteria were submitted to an orthopedic follow-up evaluation by a pediatric orthopedic expert in this field (OP). During the visit, the following medical records were collected: phases and timing of motor development (with particular attention to crawling, first steps, autonomous walking, and running); lower limb pain during walking or physical activity; orthopedic (scoliosis, valgus knee, flat foot, club foot) or other comorbidities; risky postural behaviors in childhood (such as incorrect lower-extremity swaddling); and protective factors such as the use of the baby carrier or baby sling.

Subsequently, an orthopedic objective examination was performed, including the walking test to assess any limping and/or pain and/or tiptoe walking, the Galeazzi test to detect possible differences in the length of lower limbs, and the hip joint range of motion evaluation, focusing on potential abduction or extra-rotation deficits and asymmetries. A clear asymmetric extra-rotation and/or abduction of the hip, or otherwise a range of motion (ROM) less than 60°, were considered abnormal. For ethical reasons, given the risks associated with radiation exposure, especially in the pediatric population, only patients who presented with abnormalities on the physical examination associated with a positive medical history were referred for anteroposterior (AP) pelvis and frog lateral radiographs.

### 2.4. Statistical method

For continuous variables, the Kolmogorov–Smirnov test was used to assess whether the distribution was normal or not. Categorical variables were reported as count and percentage. Continuous variables with normal distribution were expressed as mean and standard deviation; data with skewed distribution were expressed as median with interquartile range (IQR 25%–75%). Statistical analysis was performed using IBM SPSS Statistics 25.0 software (IBM Corporation, Armonk, NY, United States).

## 3. Results

### 3.1. Study population

Fifty-five patients met the inclusion criteria and underwent orthopedic evaluation (Table 1). Overall, 29 patients (52.7%) were female, and 35 (63.6%) were born by cesarean delivery; the median gestational age was 38.57 weeks. Regarding risk factors for DDH, 29 (52, 7%) patients had breech position, 23 (41, 8%) had a positive family history, and 10 (18.2%) had multiple gestations, the remaining characteristics are shown in Table 1. Hip ultrasound features are shown in Table 2. The median age of the first ultrasound was 79 days. According to Graf's classification, of left hips, 45 (81.8%) were class 1a and 10 (18, 2%) were class 1b. About right hips, 38 (69, 1%) were class 1a and 17 (30, 9%) were class 1b.

**Table 1:** Demographics and prenatal characteristics of study population.

Characteristics	Study population (*N* = 55)
Female, *n* (%)	29 (52.7%)
Cesarean section, *n* (%)	35 (63.6%)
Gestational age, *median IQR*	38.57 (2.71)
Podalic presentation after 24 weeks of gestational age, *n* (%)	29 (52.7%)
Familiarity, *n* (%)	23 (41.8%)
Multiple gestation, *n* (%)	10 (18.2%)
Prematurity, *n* (%)	13 (23.6%)
Transverse presentation, *n* (%)	2 (3.6%)
Oligohydramnios, *n* (%)	3 (5.5%)
Plagiocephaly, *n* (%)	2 (3.6%)
Comorbidities, *n* (%)	10 (18.2%)

**Table 2:** Characteristics of echographic examinations.

Characteristics	Study population (*N* = 55)
Age (days) at the time of the first echography, *median (IQR)*	79 (17)
Right hip alpha angle, *mean (SD)*	65.58 (5.52)
Right hip beta angle, *mean (SD)*	52.2 (5.75)
Right hip Graf classification, *n* (%)	
*1a*	38 (69.1%)
*1b*	17 (30.9%)
Left hip alpha angle, *mean (SD)*	66.33 (5.65)
Left hip beta angle, *mean (SD)*	50.45 (6.62)
Left hip Graf classification, *n* (%)	
*1a*	45 (81.8%)
*1b*	10 (18.2%)

### 3.2. Postural behaviors and motor development

Postural behaviors and motor development stages are shown in Table 3. Seven (12.7%) children used the baby carrier during childhood, with a median duration of 4 months. For two patients (3.6%) risky postural behaviors were found. The median age of gait onset was 13 months. One patient (1.8%), due to comorbidity (psychomotor delay), had a late gait. Two patients (3.6%) complained of balance disorders. For two patients (3.6%) parents reported changes in running.

**Table 3:** Postural risky behaviors and motor development stages with its characteristics.

Characteristics	Study population (*N* = 55)
Pouch utilization, *n* (%)	7 (12.7%)
Duration (months) of pouch utilization, *median (IQR*)^a^	4 (16)
Risky postural behaviors, *n* (%)	2 (3.6%)
**Type of risky postural behaviors, *n* (%)^b^**	
Tight bandages	1 (50%)
Let the child load the weight on his legs before he walks independently	1 (50%)
Crawling, *n* (%)	50 (90.9%)
Beginning of crawling age (months), *median (IQR)*	9 (2)
First steps, *n* (%)	55 (100%)
First steps age (months), *median (IQR)*	13 (3)
Problems in crawling and/or first steps, *n* (%)	1 (1.8%)
Not normal deambulation, *n* (%)	2 (3.6%)
Not normal run, *n* (%)	2 (3.6%)
Pain during physical activity/play, *n* (%)	6 (10.9%)

^a^ Performed on seven patients.

^b^ Performed on two patients.

### 3.3. Orthopedic follow-up examination

The median age of the children who underwent an orthopedic follow-up examination was 45 months, as shown in Table 4. At the end of the visits, a clear deficit of abduction/extra-rotation was documented for only three patients (5.5%), who underwent diagnostic investigation by radiography. However, none of the three x-rays was pathological.

**Table 4:** Characteristics of follow-up evaluation.

Characteristics	Study population (*N* = 55)
Follow-up (months), *median (IQR)*	45 (4)
Lameness, *n* (%)	0
March on the toes, *n* (%)	2 (3.6%)
Lower limb dysmetria, *n* (%)	0
Pain during deambulation, *n* (%)	0
Lower limb abduction deficit, *n* (%)	3 (5.5%)
Severe anteversion defect, *n* (%)	2 (3.6%)
Severe retroversion defect, *n* (%)	0
Radiography, *n* (%)	3 (5.5%)
Pathological radiography, *n* (%)	0

## 4. Discussion

To our knowledge, this is the first study investigating late DDH in Italy. In our population, through a clinical examination performed by an expert specialist, months after the normal ultrasound screening, we have not found any cases of late DDH.

Late DDH emerged recently, especially in US literature. Imrie et al*.* first conducted a 5-year retrospective study (16). The authors performed in breech patients, clinical and radiological examinations after 4–5 months of a negative hip ultrasound (Harcke dynamic method). They observed that among 131 breech newborns, 29% of patients had hip dysplasia in follow-up evaluation. Similarly, Busalis et al*.* (17) have found, in a small sample of 47 breech newborns, five patients with bilateral hip dysplasia to 6-months' radiographic follow-up after negative ultrasound screening (Graf technique). In addition, a UK prospective study (18) documented, in a sample of 90 breech newborns with initial negative ultrasound examination (Harcke dynamic method), an overall rate of radiographic dysplasia at 13 months equal to 7.4%. Morris et al*.* (19), through a prospective study, reported a rate of 2.2% radiographic dysplasia in breech babies at 12 months follow-up after a negative hip ultrasound. Another US study (20) reported a prevalence of late DDH of 10%. Recently, a Korean study (21) demonstrated that among 292 breech infants, two patients (0.7%) had delayed radiographic hip dysplasia at 12–24 months' age after normal ultrasound screening. Pan et al*.* (22) also reported two cases of late presenting DDH in breech infant girls after normal hip ultrasound performed at 6 weeks of age.

These studies reported very different data about the late DDH incidence, varying from 0.7% to 29%. In this regard, we can note that these are very heterogeneous studies. We can highlight different study designs and sample sizes. We can also observe different ultrasound methods, in some studies the Graf technique, in others, the dynamic Harcke method was used. In addition, neither the experience and training nor the number of sonographers who had conducted the initial ultrasound screening is always specified. Not even the follow-up is uniform, having been carried out at different times ranging from 4 to 6 months after the initial assessment up to 24 months of age. Finally, there are different criteria as well for the radiographic definition of dysplasia. However, despite the differences and limitations found, all these mentioned studies agreed about the need to implement specific follow-ups (clinic and/or radiographic) in children with breech presentation, even after a normal ultrasound examination at 6 weeks of age.

In our third-level center, children are subjected to universal screening, but following a normal ultrasound examination, even in the presence of risk factors, no subsequent follow-up is recommended. In the light of the evidence mentioned above, we conducted this study to investigate the need for additional controls in patients with risk factors. We have not been limited to breech babies, but we have analyzed the patients with other risk conditions such as familiarity, oligohydramnios, and multiple pregnancies. Walking children were subjected to orthopedic clinical evaluation and, in the case of an objective pathological examination, to radiological investigation. No case of late DDH has been found in our population. A possible explanation for this result could be the ultrasound method. One of the main problems concerning ultrasound is that this examination is highly operator dependent. Moreover, several techniques have been described to evaluate hips in newborns [where the Graf classification is the most widely accepted technique (12)], but they are not always reproduced correctly (26). The initial studies report a greater incidence of late DDH that is reduced in subsequent studies. In consideration of the increasing attention given to the execution of technically correct ultrasound scans, it could therefore be speculated whether the cases of dysplasia identified during follow-up, especially in the initial studies, were really “late DDH” or instead “late-detected DDH.” One of the strengths of our study concerns the ultrasound method. In fact, in our center, a single certified (hip ultrasound training by Graf) operator with many years of experience carries out all ultrasound scans, paying close attention to the production of the correct standard section. Another possible hypothesis is related to postural measures. In fact, we investigated the potential presence of risky or protective behaviors at follow-up visit and only in two cases did we find risky behaviors. Because DDH is a dynamic pathological process, the hip joint could be susceptible even in the postnatal period to mechanical stresses deriving from incorrect postural conduct. The previously mentioned studies do not provide information regarding any incorrect postural behaviors in their study population. Another possible explanation is related to follow-up evaluation. In fact, although our follow-up clinical examination was performed by a pediatric orthopedic specialist, highly qualified and with years of experience in this field, radiograph was not performed for all patients. Given the risk related to radiation exposure, especially in the pediatric population, and considering the area to be explored (which contains the gonads), for ethical reasons, the authors decided to not perform a radiograph in case of a completely negative objective examination. Therefore, our main limit is that results are based exclusively on clinical evaluation rather than radiological assessments. Additional study's limitations are a single-center study and a relatively small sample size.

## 5. Conclusions

Our study did not reveal cases of late DDH during the clinical follow-up aimed at children with risk factors for DDH after an initial negative screening ultrasound examination (performed by an experienced and certified operator). However, given the consequences of not diagnosing DDH, further studies with a wider population, a longer follow-up and a control group are needed to determine whether it is necessary to change the current management of patients with risk factors for DDH, especially in breech newborns.

## Data Availability

The original contributions presented in the study are included in the article/Supplementary Material, further inquiries can be directed to the corresponding author.
